# Proliferation Pattern of Pediatric Tumor-Derived Mesenchymal Stromal Cells and Role in Cancer Dormancy: A Perspective of Study for Surgical Strategy

**DOI:** 10.3389/fped.2021.766610

**Published:** 2021-11-04

**Authors:** Gloria Pelizzo, Federica Riva, Stefania Croce, Maria Antonietta Avanzini, Gloria Acquafredda, Annalisa de Silvestri, Emanuela Mazzon, Placido Bramanti, Gianvincenzo Zuccotti, Giuliano Mazzini, Valeria Calcaterra

**Affiliations:** ^1^Pediatric Surgery Department, “Vittore Buzzi”, Children's Hospital, Milan, Italy; ^2^Department of Biomedical and Clinical Science, “L. Sacco”, University of Milan, Milan, Italy; ^3^Department of Public Health, Experimental and Forensic Medicine, Histology and Embryology Unit, University of Pavia, Pavia, Italy; ^4^Immunology and Transplantation Laboratory, Cell Factory, Pediatric Hematology Oncology Unit, Department of Maternal and Children's Health, Fondazione IRCCS Policlinico S. Matteo, Pavia, Italy; ^5^Biometry and Clinical Epidemiology, Scientific Direction, Fondazione IRCCS Policlinico San Matteo, Pavia, Italy; ^6^IRCCS Centro Neurolesi “Bonino-Pulejo”, Messina, Italy; ^7^Pediatric Department, “Vittore 86 Buzzi”, Children's Hospital, Milan, Italy; ^8^Istituto di Genetica Molecolare-Centro Nazionale delle Ricerche (IGM-CNR), Pavia, Italy; ^9^Pediatrics and Adolescentology Unit, Department of Internal Medicine, University of Pavia, Pavia, Italy

**Keywords:** mesenchymal stromal cells, proliferation pattern, cell cycle, tumor, children

## Abstract

The explanation for cancer recurrence still remains to be fully elucidated. Moreover, tumor dormancy, which is a process whereby cells enter reversible G_0_ cell cycle arrest, appears to be a critical step in this phenomenon. We evaluated the cell cycle proliferation pattern in pediatric tumor-derived mesenchymal stromal cells (MSCs), in order to provide a better understanding of the complex mechanisms underlying cancer dormancy. Specimens were obtained from 14 pediatric patients diagnosed with solid tumors and submitted to surgery. Morphology, phenotype, differentiation, immunological capacity, and proliferative growth of tumor MSCs were studied. Flow cytometric analysis was performed to evaluate the cell percentage of each cell cycle phase. Healthy donor bone marrow-derived mesenchymal stromal cells (BM-MSCs) were employed as controls. It was noted that the DNA profile of proliferating BM-MSC was different from that of tumor MSCs. All BM-MSCs expressed the typical DNA profile of proliferating cells, while in all tumor MSC samples, ≥70% of the cells were detected in the G0/G1 phase. In particular, seven tumor MSC samples displayed intermediate cell cycle behavior, and the other seven tumor MSC samples exhibited a slow cell cycle. The increased number of tumor MSCs in the G0–G1 phase compared with BM-MSCs supports a role for quiescent MSCs in tumor dormancy regulation. Understanding the mechanisms that promote dormant cell cycle arrest is essential in identifying predictive markers of recurrence and to promote a dedicated surgical planning.

## Introduction

The overall incidence rates of childhood cancer vary between 50 and 200 per million children across the world (1). A thorough study on this issue revealed an incidence rate of 138.5 children in every 1 million children worldwide (2). However, cancer is the third leading cause of death among children ages 1–4 and the second leading cause of death among children age 5–14 (3), representing about 8% of all pediatric deaths (4–6). Indeed, after surgical and medical treatment, recurrence, or cancer relapse after an initial diagnosis has been frequently recorded (4–6).

A proposed mechanism underlying the persistence of covert cancer cells during and after treatment is that some cancer stem cells enter a reversible quiescent or dormant state in which they are relatively resistant to radiation and chemotherapy. Conventional chemotherapy regimens include DNA-damaging agents and spindle poisons, and their effect is, therefore, dependent on the active cycling of tumoral cells through the S and M cell cycle phases, respectively. However, both cell intrinsic characteristics and extrinsic influences from surrounding normal cells determine tumor cell dormancy (7). Extrinsic factors include mesenchymal stromal cells (MSCs), endothelial cells (ECs), and immune cells that form the niche of the tumor. As described in *in vitro* leukemia models, blasts communicate closely with MSCs (8), and contact with MSCs has been demonstrated to provide key survival signals to leukemic blasts, rendering them resistant against the non-genotoxic components of leukemia treatment protocols (8, 9). Mesenchymal stromal cells play different roles in modulating tumor progression, growth, and metastasis. They are recruited to the tumor site in large numbers and subsequently have an important microenvironmental role in modulating tumor progression and drug sensitivity. However, the effects of the tumor microenvironment (TME) on MSCs remain poorly understood. It has been reported that a paracrine effect of cancer cells slows cycling and chemoresistance, through the secretion of soluble factors promoting a more stem-like state of MSCs (10). Additionally, the contact between cancer cells and MSCs in regulating cancer dormancy should not be excluded (11).

Therefore, the aim of this study was to characterize the proliferation pattern of the cell cycle in pediatric tumor-derived MSCs, in order to enhance our understanding of the complex mechanisms, implicated in the cancer dormancy process, that may influence therapeutic response.

## Materials and Methods

### Patients

Fourteen pediatric patients (eight females and six males; median age 5 years, range 9 months to 15 years), diagnosed with solid tumors (three neuroblastomas, three lymphomas, three nephroblastomas, and five others) and submitted to surgery were enrolled. Mesenchymal stromal cell isolation and expansion were performed starting from residual material for histological analysis. Samples were collected prior to chemotherapy. Stored bone marrow-derived mesenchymal stromal cells (BM-MSCs), obtained as previously described (12) from healthy donors (two females and two males; median age 5.5 years, range 4–7 years) enrolled for hematopoietic stem cell donation, were used as a control group.

The study was performed according to the Declaration of Helsinki and with the approval of the Institutional Review Board of the Children's Hospital “G. Di Cristina” (registry number 87 Civico 2017). Informed written consent was obtained from the parents and/or legal guardian after receiving information about the study.

### Methods

#### Tumor Mesenchymal Stromal Cell Isolation and Expansion

Tumor tissue was mechanically dissociated and treated with collagenase type II as previously described (11). Tumor MSCs were expanded following the procedure normally used for BM-MSCs (12). Briefly, cells were plated in flasks or wells (Corning Costar, Corning, NY, USA) according to the cell number obtained, at a density of 160,000/cm^2^ in complete medium [D-MEM + GlutaMAX (Gibco), supplemented with 10% FBS (Euroclone), 50 mg/ml of gentamicin, and 1% penicillin (Sigma Aldrich)] and cultured at 37°C, 5% CO_2._

Culture medium was changed twice a week until ≥80% confluence was reached; then tumor MSCs were trypsinized (Trypsin EDTA, Euroclone) and replated at a density of 4,000 cells/cm^2^ for expansion (12). Cells were propagated to reach senescence.

#### Characterization of *ex vivo* Expanded Tumor Mesenchymal Stromal Cells

As defined by the Mesenchymal and Tissue Stem Cell Committee of the International Society for Cellular Therapy (ISCT), MSCs must be plastic adherent and exhibit a spindle-shape morphology in standard culture conditions. Proliferative capacity was evaluated as cumulative population doubling (cPD) resulting from the sum of PD at each passage calculated with the following formula PD = log_10_ (no. of harvested cells/no. of seeded cells)/log_10_2.

Tumor MSCs were characterized by flow cytometry, using fluorescein isothiocyanate (FITC)- or phycoerythrin (PE)-labeled monoclonal antibodies specific for surface antigens: CD73, CD34, CD90, CD14, CD45, CD31, CD105, class I-HLA, and HLA-DR (Beckman Coulter, IL, Milan, Italy), as previously described (10). Analysis was performed by direct immunofluorescence with a FACS Navios flow cytometer (Beckman Coulter).

Tumor MSCs were cultured in osteogenic differentiation induction medium [αMEM, 10% FBS, 10^−7^ M dexamethasone, 50 mg/ml of L-ascorbic acid, and 5 mM β-glycerol phosphate (all from Sigma-Aldrich)], and in adipogenic differentiation medium [αMEM, 10% FBS, 10^−7^ M dexamethasone, 50 mg/ml of L-ascorbic acid, 5 mM β-glycerol phosphate, 100 mg/ml of insulin, 50 mM isobutyl methylxanthine (all from Sigma-Aldrich), and 0.5 mM indomethacin (MP Biomedica)]. The medium was replaced twice a week. After 21 days of culture, osteogenic differentiation was assessed by staining for alkaline phosphatase (AP) activity with Fast Blue and for calcium deposition, with Alizarin Red S stain (both from Sigma-Aldrich), while adipogenic differentiation was demonstrated by staining of fat droplets with Oil Red O (Bio Optica, Milan, Italy).

Tumor MSC senescence was defined by the β-galactosidase (SA-β-gal) staining Kit (Cell Signaling Technology, Danvers, MA, USA), according to the instructions of the manufacturer. The evaluation of senescence was performed by bright-field microscopy.

#### DNA Staining for Cell Cycle Cytometric Analysis

Tumor MSCs at P3–P4, were collected after trypsinization. Cell suspensions were centrifuged at 1,200 rpm for 5 min, and then pellets were rinsed twice with phosphate-buffered solution (PBS). After the last centrifugation, 1 × 10^6^ cells were resuspended in 2 ml of DNA staining solution (50 μg/ml of propidium iodide in PBS, 0.1% Igepal, 100 U/ml of RNase type 1A; all reagents from Sigma-Aldrich) and left for 2 h at room temperature before measurement. For the four MSC samples at P3–P4, from healthy hematopoietic stem cell donor (HD), bone marrow was used as a control group.

The cell percentage at each cell cycle phase was evaluated by flow cytometry, as previously described (13). Monoparametric conventional analysis was performed with a Partec PAS II flow cytometer (Sysmex, Milan) using a blue laser and with data recorded on a dedicated computer integrated in the system.

To ensure the best instrumental analytical performance, preliminary alignment, and control were always set up using standard calibration fluorescence beads (Sysmex Ref-4018 KW 160317). The best histogram resolution was achieved measuring a minimum of 50,000 cells. All measurements were performed blindly.

To excite and intercalate propidium iodide into the double-stranded nuclear DNA, the laser line was set at 488 nm, while a 610-nm-long pass filter permitted the selection and measurement of the red fluorescence emission. Data analyses were displayed as frequency histograms of red fluorescence intensities (equivalent to DNA content). Cell cycle analysis and estimation of the three G0/G1, S, and G2/M phases were analyzed with FlowMax software. Cell number in all phases was expressed as cell percentage frequency.

#### Statistical Analysis

Qualitative variables were described as count and percentage. Comparison between BM-MSCs and tumor MSCs was performed by Fisher exact test. Values of *p* < 0.05 were considered statistically significant. Analyses were performed using the SPSS statistical package (SPSS, Chicago, IL, USA) and Stata 8.0.

## Results

### Features of the Tumor-Derived MSCs

Residual material from solid pediatric tumor biopsies taken for histological analysis was used as the starting material. In each case, MSC expansion was possible, and all MSC cultures met the minimal criteria defined by the ISCT (14).

**Figure 1:**
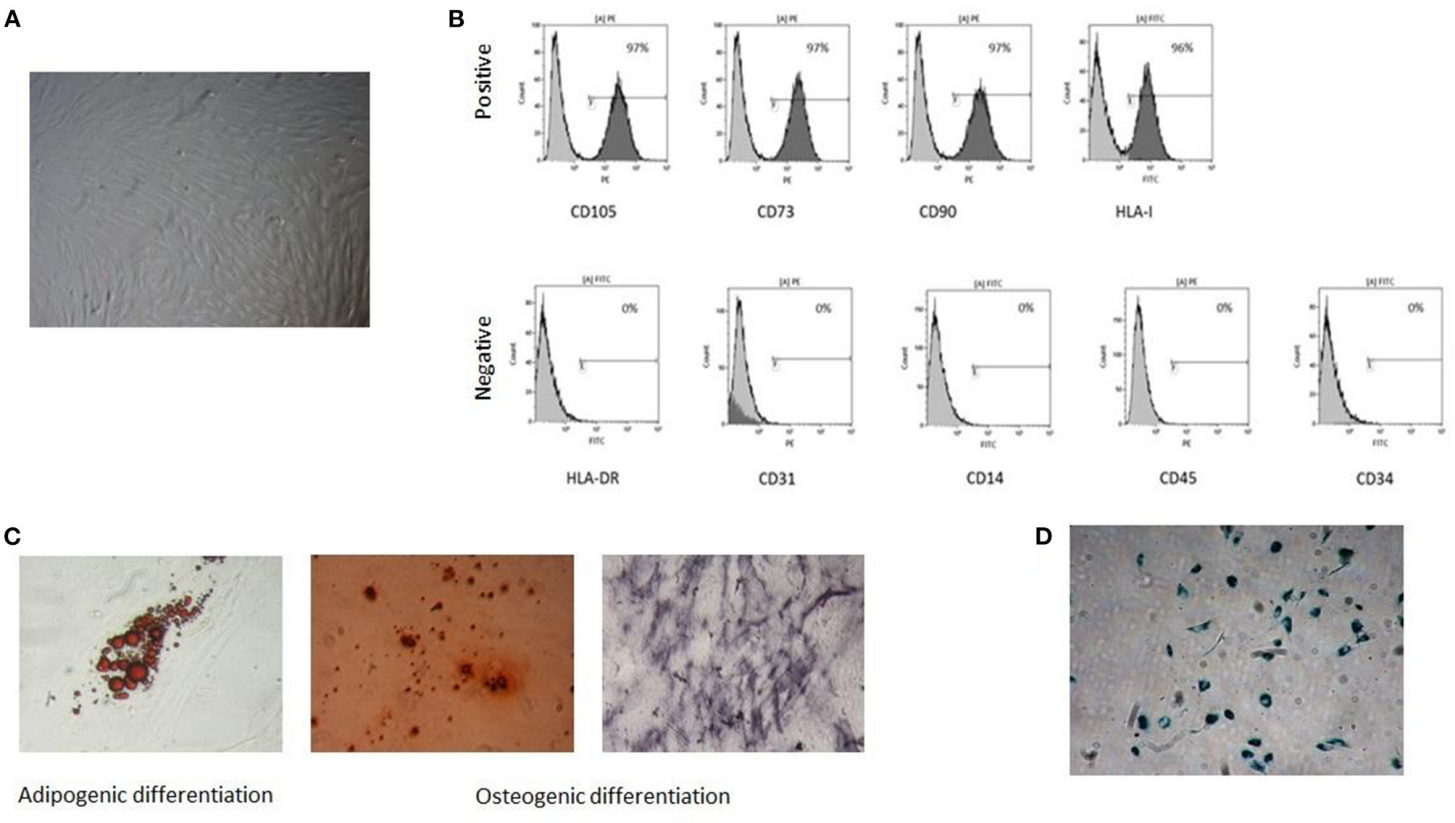
Characterization of tumor mesenchymal stromal cells (MSCs). A representative sample is reported in the figure: **(A)** spindle-shape morphology, **(B)** immunophenotype with positive and negative surface antigens, **(C)** *in vitro* adipogenic and osteogenic differentiation capacity, **(D)** senescent tumor MSC at P13.

As preliminarily reported for NB-derived MSCs (NB) (11), tumor MSCs exhibit spindle-shape morphology and are plastic adherent (Figure 1A). Our tumor MSCs expressed the following surface antigens: CD73, CD90, CD105, and HLA-class I ≥95% and CD34, CD14, CD45, CD31, and HLA-DR ≤ 5% (Figure 1B). They presented the capacity to differentiate into osteoblast and adipocytes (Figure 1C). Senescence was reached at a median passage of P11 (range P6–P23) (Figure 1D).

Tumor MSCs did not exhibit any differences in phenotypical or functional characteristics among the different kinds of tumors.

### Cell Cycle Analysis by Flow Cytometry on Tumor Mesenchymal Stromal Cells

**Figure 2:**
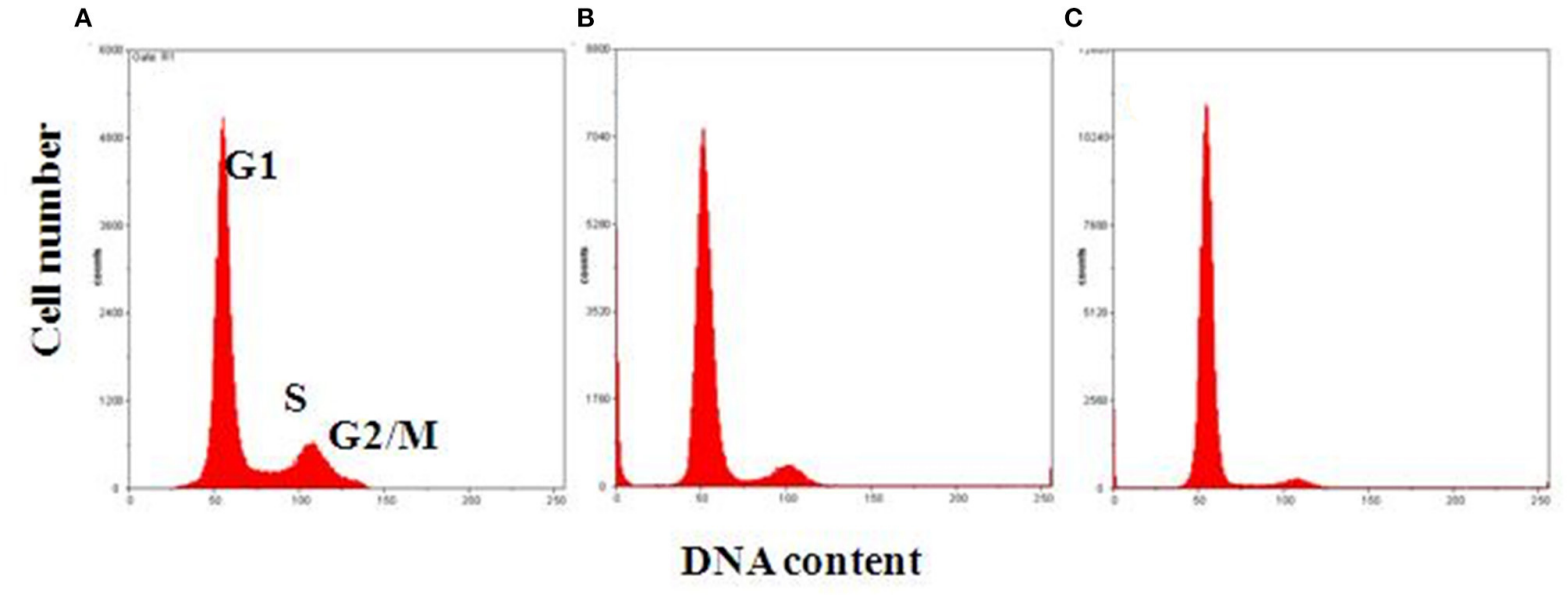
Representative flow cytometric analysis of MSC DNA profiles. Histograms show cell cycle progression in G1, S, and G2/M phases for each representative condition. **(A)** Proliferating cells with 60% of cells in G1, 20% in S, and 20% in G2/M phase. **(B)** Intermediate condition of proliferation, with 70% of cells in G1, 15% in S, and 15% in G2/M phase. **(C)** Slow cell proliferation, with 85% of cells in G1, 5% in S, and 10% in G2/M phase on the x-, y-axes, respectively: DNA content vs. cell number.

Blindly evaluated data related to MSC DNA content allowed us to define three different classes of cell cycle behavior. Proliferating samples were defined as cells at the following concentrations: 55–65, 20–25, and 20%, respectively, in G0/G1, S, G2/M. Samples were considered to be in an intermediate cell cycle condition when 70–75% of cells were observed in G1, 10–15% in S, and 10–15% in G2/M. A slow cell cycle had a high cell number in the G1 phase (80–90%) and lower cell percentage in S (5–10%) and G2/M (10–15%) phases, respectively (Figure 2; Table 1).

**Table 1:** Definition of the three classes of cell cycle behavior.

**Proliferation pattern**	**Percentage of cells in G0/G1**	**Percentage of cells in S**	**Percentage of cells in G2/M**
Proliferating	55–65	20–25	20
Intermediate	70–75	10–15	10–15
Slow	80–90	5–10	10–15

**Figure 3:**
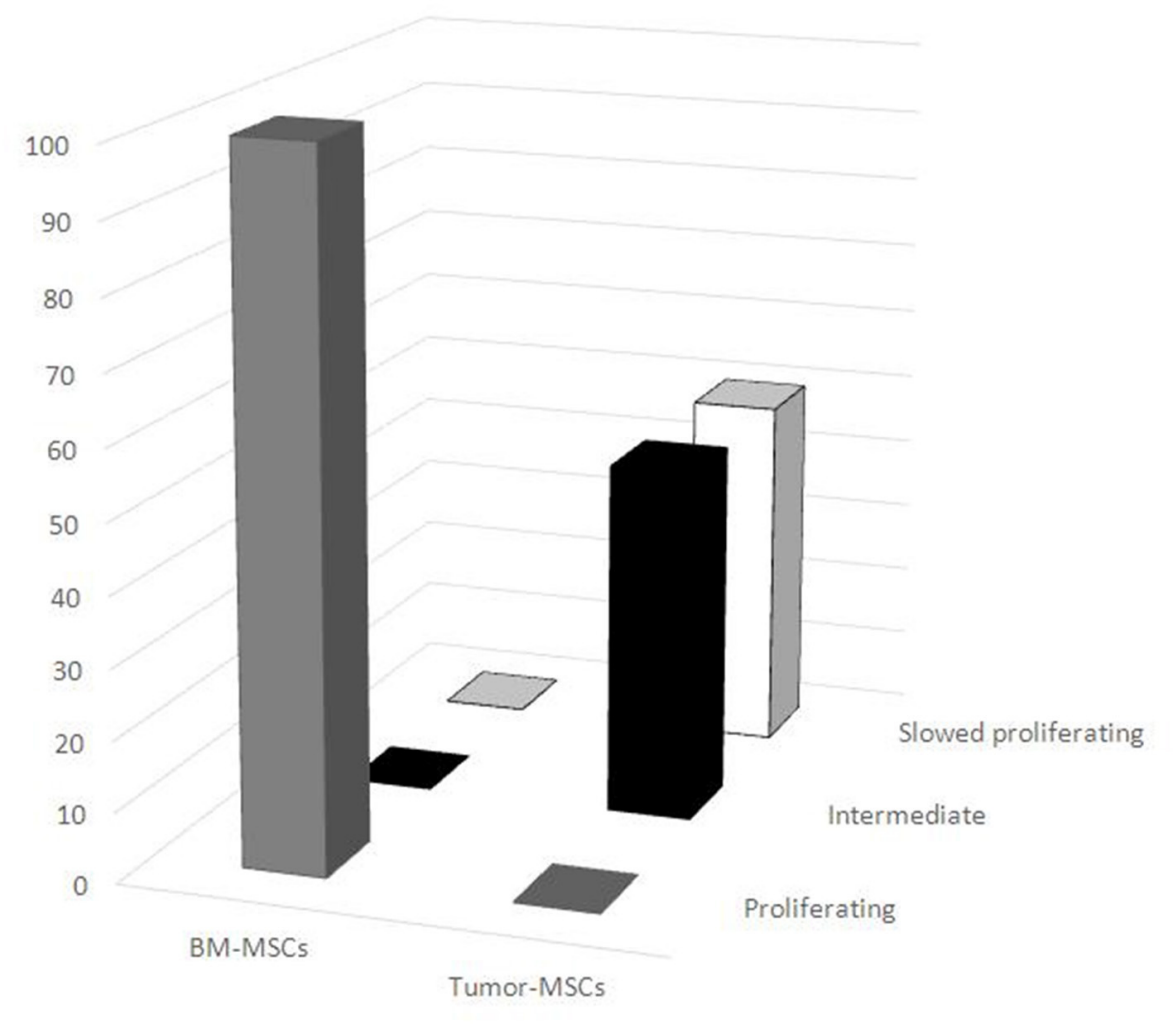
Distribution of proliferating mesenchymal stromal cells (MSCs). Different DNA proliferating cell profiles were noted between BM-MSCs and tumor MSCs. All BM-MSCs expressed the typical DNA profile of proliferating cells, while all tumor MSC samples had a higher percentage of cells detected in the G0/G1 phase. BM-MSC, bone marrow-mesenchymal stromal cells; tumor MSCs, tumor-derived mesenchymal stromal cells.

Different proliferating cell DNA profiles were noted when comparing BM-MSCs with tumor MSCs. All of the BM-MSCs had a typical DNA profile of proliferating cells, while all tumor-MSC samples had ≥70% of cells detected in the G0/G1 phase. In particular, seven tumor MSC samples (one neuroblastoma, two lymphomas, and four others) displayed intermediate behavior, and the other seven tumor MSC samples (two neuroblastomas, two nephroblastomas, one lymphoma, and one other) were in a slow cell cycle. The distribution of proliferating BM-MSCs and tumor MSCs resulted significantly different between the two groups (*p* < 0.001) (Figure 3).

## Discussion

Tumor relapse and metastasis in some cancers can arise years or decades after initial surgical and medical treatment and are responsible for the majority of cancer-related deaths (15). The identification of predictive markers for recurrence should be crucial to identify a correct surgical strategy. Cancer recurrence has not been fully elucidated. Moreover, tumor dormancy seems to be a critical condition in this phenomenon.

Cancer cell dormancy is defined as a process in which cells enter reversible G_0_ cell cycle arrest (16), called quiescence. Quiescent cells may acquire additional mutations, survive in a new environment and initiate metastasis, become resistant to chemotherapeutic drugs, and evade immune destruction, thereby influencing cancer progression (16). Different factors have been suggested as contributors to cell dormancy, including complex interactions between metastatic cells and the microenvironment (17–19).

The TME includes endothelial cells, fibroblasts, MSCs, and various immune cells, which are together with cytokines and growth factors embedded in the tumor stroma endowed with specific physical and biomechanical cues (20). It is widely accepted that MSCs participate in each step of tumor development, including relapse and metastasis, due to tumor-homing ability, dynamic phenotype, and immunoregulatory activity (21). It has been reported that tumor MSCs may be driven by the tumor secretome determining their molecular and functional behavior. *In vitro* tumor MSC angiogenic capacity and tumor growth have been described to be supported by melanoma cells. On the contrary, glioblastoma cells reduced protumorigenic effect (22). Moreover, medullary thyroid carcinoma, human breast carcinoma, and glioblastoma cells determined changes in MSCs, stimulating their inhibitory effect on cell proliferation and tumor growth (23–25).

Several cells, including MSCs, are recruited to the stroma of tumors where they acquire important microenvironmental roles in modulating tumor progression and drug sensitivity (26–28). Even though clear evidence has not been reported, it is known that the reaction of the host to tumors includes the release of proinflammatory cytokines and chemokines, which modulate the TME, support tumor growth, invasion, and metastasis (29–33). However, the crosstalk between tumor and tumor MSCs is sustained by a more complex pattern determined also by the tumor nature (34). It has been shown that tumor cells secreted several factors exerting different effects on the MSC activity and molecular changes bringing alterations in capacity to stimulate tumor growth (22). El-Badawy et al. (10) have recently reported that co-culture of tumor cell lines with BM-MSCs resulted in their phenotypic and functional alteration.

Additionally, regulation and modulation of the cytokine repertoire produced by various tissue-derived MSCs may affect the cancer cell cycle. Fathi et al. (35), reported that cell cycle progression of K562 co-cultured with BM-MSCs resulted in an accumulation of cells in the G0/G1 phase, with slowed entry into the S phase. Fonseka et al. (36) demonstrated that the arrest of K562 growth in the G0/G1 phase was due to the anti-proliferative effect of human umbilical cord blood-derived MSCs.

The role of cancer cells in modulating the cell cycle of MSCs derived from the TME has not been previously considered. A better knowledge of the mechanisms that promote dormant cell cycle arrest could allow to identify new perspectives of study in pediatric surgery. A dedicated surgical strategy for the prevention of cancer recurrence could be defined. In the present study, we noted that tumor MSCs represent a population with phenotypical and functional characteristics of BM-MSCs, with a slow or intermediate cell cycle.

Considering the hypothesis that cytokines and growth factors might be highly involved in the anti-tumor effect mediated by MSCs, a high content of MSCs blocked in the G1 phase, as observed in our study, supports a scenario of “selective” cytokine secretion able to regulate tumor cell arrest in the G0/G1 phase, thereby inducing cancer dormancy (37). As documented by Li et al. (38), senescent MSCs may alter the tissue microenvironment and affect nearby malignant cells via cytokine secretion. In the same way, quiescent MSCs could have tumor-regulating effects.

Additionally, as reported by El-Badawy et al. (10), similar to cancer-induced stem cells, tumor-derived MSCs are slow cycling upon exposure to cancer cell-secreted factors. However, further studies are mandatory to support these hypotheses.

We recognize that this study has some limitations. The sample size was small, but the results were consistent with those of other studies investigating this specific population. The study was limited to characterizing pediatric tumor-derived MSCs, without investigation of the regulation mechanisms involved in cancer cellular processes. Additionally, our cases were not age-matched with controls; as reported (39), the same and identical results were found irrespective of whether matching or not matching was applied. Thus, it is possible that age and underlying condition may affect the outcomes studied. Finally, the follow-up of patients was not adequate to provide any correlation between the tumor MSC effect and tumor prognosis.

Despite these limitations, our data support a role for tumor MSCs in the cross talk between cancer cells and their microenvironment and promotion of cancer dormancy.

In conclusion, we characterized the proliferation pattern of pediatric tumor-derived MSCs. The increased number of tumor MSCs in the G0–G1 phase compared with BM-MSCs supports the role of quiescent MSCs in tumor dormancy regulation. Further studies focusing on the mechanisms, which enable a dormant cell cycle, are needed.

New predictive markers of risk recurrence should be evaluated as an innovative perspective of surgical strategy in children.

## Data Availability Statement

The raw data supporting the conclusions of this article will be made available by the authors, without undue reservation.

## Ethics Statement

The studies involving human participants were reviewed and approved by Istitutional Review Board of the Children's Hospital G. Di Cristina (registry number 87 Civico 2017). Written informed consent to participate in this study was provided by the participants' legal guardian/next of kin.

## Author Contributions

GP, FR, MA, GM, and VC conceptualized the study. GP, FR, SC, MA, GA, EM, AS, PB, GZ, GM, and VC developed the methodology. AS performed the formal analysis. SC, MA, and GA were in charge of the investigation. GP, FR, MA, SC, GA, AS, EM, PB, GM, and VC prepared and wrote the original draft. GP, FR, MA, EM, PB, GZ, GM, and VC wrote, reviewed, and edited the manuscript. GP, FR, MA, EM, PB, GZ, GM, and VC supervised the study. All authors have read and agreed to the published version of the manuscript.

## Conflict of Interest

The authors declare that the research was conducted in the absence of any commercial or financial relationships that could be construed as a potential conflict of interest.

## Publisher's Note

All claims expressed in this article are solely those of the authors and do not necessarily represent those of their affiliated organizations, or those of the publisher, the editors and the reviewers. Any product that may be evaluated in this article, or claim that may be made by its manufacturer, is not guaranteed or endorsed by the publisher.
